# Effects of *Cudrania tricuspidata* and *Sargassum fusiforme* extracts on hair growth in C57BL/6 mice

**DOI:** 10.1186/s42826-023-00154-7

**Published:** 2023-02-17

**Authors:** Priyanka Rajan, Premkumar Natraj, Nak Hyoung Kim, Jae-Hoon Kim, Hyuk Joon Choi, Chang-Hoon Han

**Affiliations:** 1 Department of Biochemistry, College of Veterinary Medicine, Jeju National University, Jeju, 63243 Republic of Korea; 2 BK Bio, Jeju, 63359 Republic of Korea

**Keywords:** *Cudrania tricuspidata*, *Sargassum fusiforme*, Anagen factors, Catagen-telogen factors, Hair growth, RNA sequencing analysis

## Abstract

**Background:**

*Cudrania tricuspidata* is a perennial plant, and *Sargassum fusiforme* is a brown seaweed with numerous potential benefits, including anticancer, anti-inflammatory, and antioxidant activities. However, the efficacies of *C. tricuspidata* and *S. fusiforme* on hair growth have not yet been elucidated. Therefore, the present study examined the effects of *C. tricuspidata* and *S. fusiforme* extracts on hair growth in C57BL/6 mice.

**Results:**

ImageJ demonstrated that drinking and skin application of *C. tricuspidata* and/or *S. fusiforme* extracts significantly increased the hair growth rate in the dorsal skin of C57BL/6 mice compared to the control group. Histological analysis confirmed that drinking and skin application of *C. tricuspidata* and/or *S. fusiforme* extracts for 21 days significantly increased the length of hair follicles on the dorsal skin of treated C57BL/6 mice compared to that in the control mice. RNA sequencing analysis revealed that hair growth cycle-related factors (anagen factors) such as Catenin Beta 1 (*Ctnnb1*) and platelet-derived growth factor (*Pdgf*) were upregulated (> twofold) only by *C. tricuspidate* extracts, whereas vascular endothelial growth factor (*Vegf)* and *Wnts* were upregulated by both *C. tricuspidata* or *S. fusiforme* applications in treated mice (compared to the control mice). In addition, oncostatin M (*Osm*, a catagen-telogen factor) was downregulated (< 0.5 fold) by *C. tricuspidata* when administered via both skin and drinking mode in treated mice compared to that in control mice.

**Conclusions:**

Our results suggest that *C. tricuspidata* and/or *S. fusiforme* extracts show potential hair growth efficacy by upregulating anagen factor genes, including *β-catenin*, *Pdgf, Vegf,* and *Wnts*, and downregulating catagen-telogen factor genes, including *Osm*, in C57BL/6 mice. The findings suggest that *C. tricuspidata* and/or *S. fusiforme* extracts *are* potential drug candidates to treat alopecia.

## Background

Although hair loss has been recognized as a series of aging phenomena, it has recently been found that hair loss progresses owing to various factors such as stress, nutritional imbalance, and chemicals, along with several genetic factors [1, 2].

Hair plays various roles in the human body, such as protecting the head from direct UV rays, maintaining the body temperature, and affecting external appearance [3]. Hair formation has a cyclical pattern characterized by phases of regeneration (anagen), regression (catagen), rest (telogen), and shedding of old hair fiber (exogen) [4]. During the anagen phase, follicles produce an entire hair shaft from the tip to the root; during catagen, telogen, and exogen, follicles reset and prepare to start the next growth phase and make a new hair shaft [5]. Several signaling pathways have been implicated in regulating hair formation in humans and mice, including Wnt/β-catenin and transforming growth factor-β/bone morphogenic protein (TGF-β/BMP) [6].

*Cudrania tricuspidata* (Moraceae) is a perennial plant with numerous medicinal and nutritional applications, including anticancer and antibacterial activity [7–10]. *Sargassum fusiforme* is a brown seaweed with numerous potential benefits, including anti-inflammatory and antioxidant activities [11]. However, the hair growth-promoting effects of *C. tricuspidata* and *S. fusiforme* on the hair growth cycle have not been established.

The present study aimed to test the hypothesis that *C. tricuspidata* and *S. fusiforme* extracts enhance hair growth in C57BL/6 mice.

## Results

### *C. tricuspidata* and *S. fusiforme* extracts improve the hair growth rate in C57BL/6 mice

**Fig. 1:**
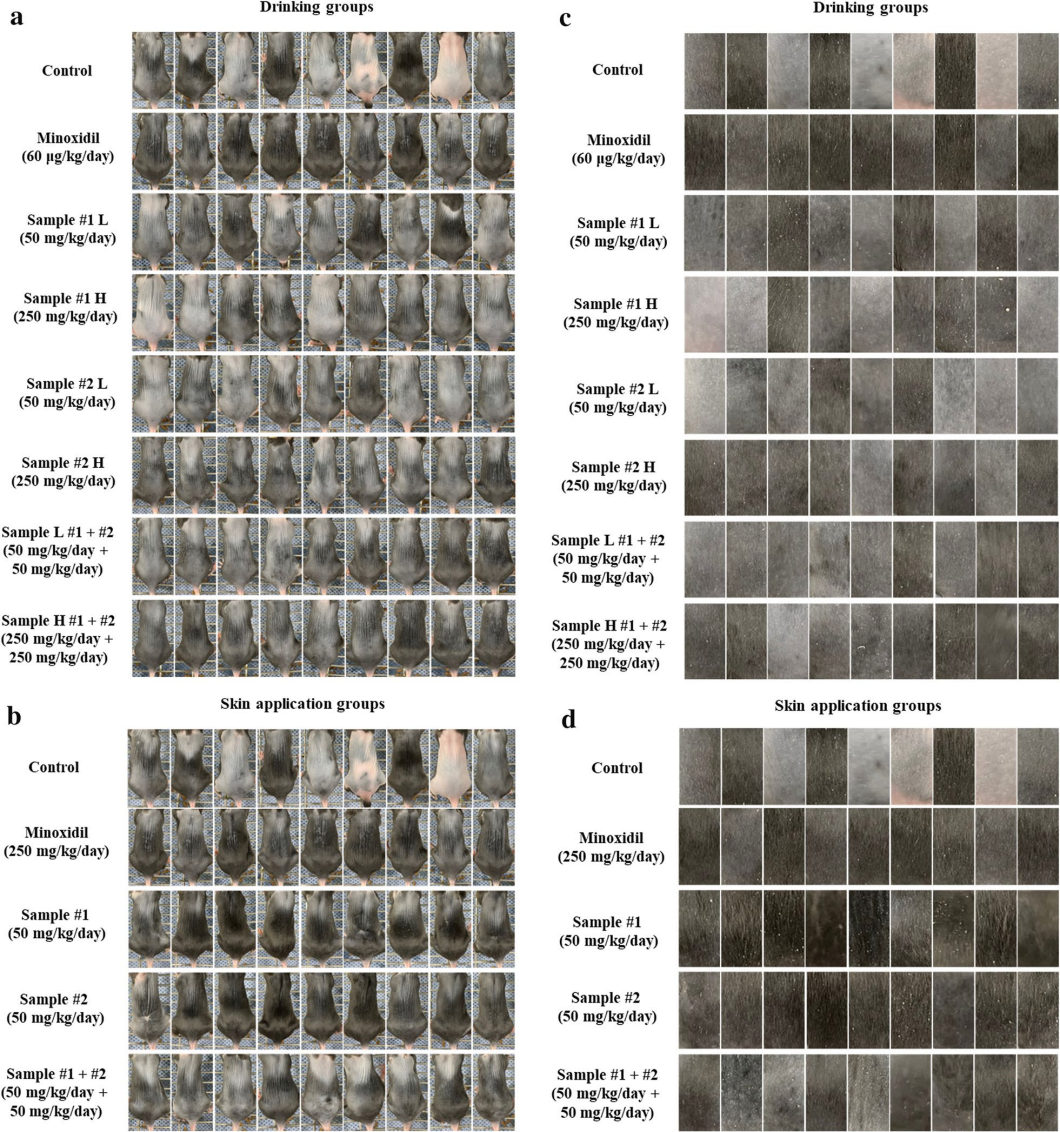
The dorsal skin of C57BL/6 mice treated with *C. tricuspidata* and/or *S. fusiforme* extracts. Images of the dorsal skin of mice in the drinking groups **a** and skin application groups **b** treated with *C. tricuspidata* and/or *S. fusiforme* on day 11. Area of the dorsal skin of mice used for Image J analysis after drinking **c** and skin application **d** of *C. tricuspidata* and/or *S. fusiforme*. Sample #1: *C. tricuspidata* and Sample #2: *S. fusiforme*; Doses of samples were applied to groups, as described in the Materials and methods section; L: Low dose and H: High dose

The efficacy of *C. tricuspidata* and *S. fusiforme* extracts with respect to hair growth was evaluated in C57BL/6 mice. Figure 1 shows pictures taken on day 11 when the difference in hair growth was most remarkable among the groups during the drinking or skin application of *C. tricuspidata* and *S. fusiforme* to C57BL/6 mice. From the images taken on day 11, the hair growth rate was evaluated using ImageJ in the drinking groups (Fig. 1c) and the skin application groups (Fig. 1d).

**Fig. 2:**
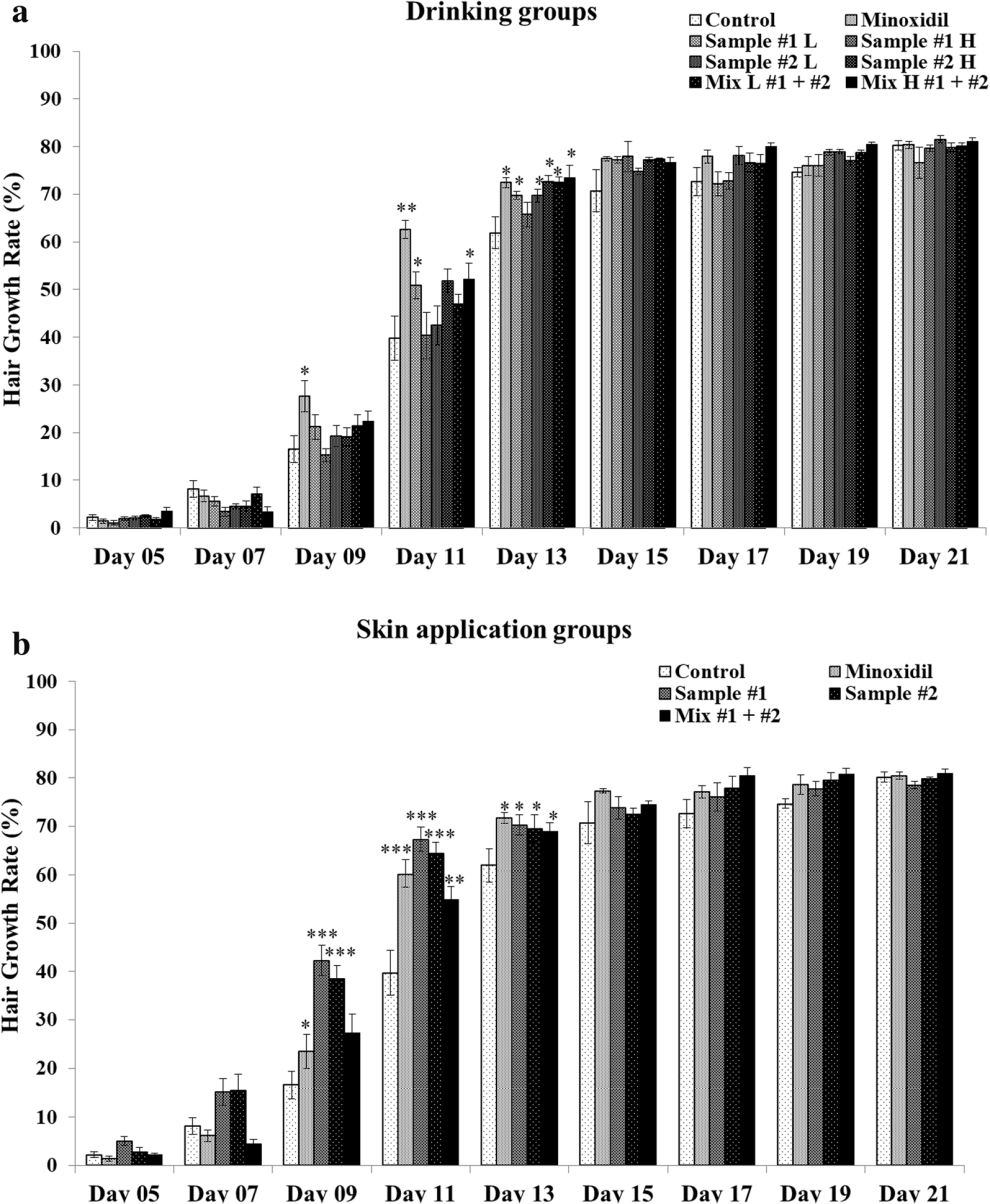
Effects of *C. tricuspidata* and/or *S. fusiforme* on hair growth rate in C57BL/6 mice. Images of the dorsal skin of the mice were captured on days 5, 7, 9, 11, 13, 15, 17, 19, and 21, and the hair growth rate was estimated using ImageJ software. Hair growth rates of the drinking groups **a** and skin application groups **b** treated with *C. tricuspidata* and/or *S. fusiforme* measured on days 5, 7, 9, 11, 13, 15, 17, 19, and 21. Sample #1: *C. tricuspidata* and Sample #2: *S. fusiforme*. Data are presented as mean ± standard error of the mean (SEM) of replications. ^*^p < .05, ^**^p < .005, ^***^p < .0005 (one-way ANOVA) compared to the control group; L: Low dose; H: High dose

The hair growth rates, which were evaluated using ImageJ analysis of the images acquired from days 5 to 21, are shown in Fig. 2. On day 9, only the minoxidil drinking subgroup showed a significant (*p* < 0.05) increase in hair growth rate compared to the control subgroup (Fig. 2a). On day 11, the hair growth rate was significantly higher in all subgroups than that in the control group; the highest difference was obtained for the minoxidil group. On day 13, the treatments demonstrated similar trends of increased hair growth rate but did not differ from that in control. On days 15 through 21, no significant differences in hair growth rate were observed (Fig. 2a).

In contrast, in the skin application subgroups, in mice treated with *C. tricuspidata* (*p* < 0.0005) and *S. fusiforme* (*p* < 0.0005) extracts, the hair growth rate was significantly increased compared to that in control mice and those treated with minoxidil on days 9 and 11 (Fig. 2b). On day 13, all subgroups in the skin application group, the hair growth rate was significantly higher in mice treated with *C. tricuspidata* and/or *S. fusiforme* (*p* < 0.05) than that in control mice (Fig. 2b). On day 15 onwards, no significant differences were observed for hair growth rate among the five subgroups.

Taken together, these results demonstrated that the supplementation of *C. tricuspidata* and/or *S. fusiforme* extracts as a drink was most effective on days 11 to 13. In contrast, the skin application of the extracts revealed the highest efficacy from days 9 to 11.

### *C. tricuspidata* and *S. fusiforme* extracts improve the hair follicle length in the dorsal skin of C57BL/6 mice

**Fig. 3:**
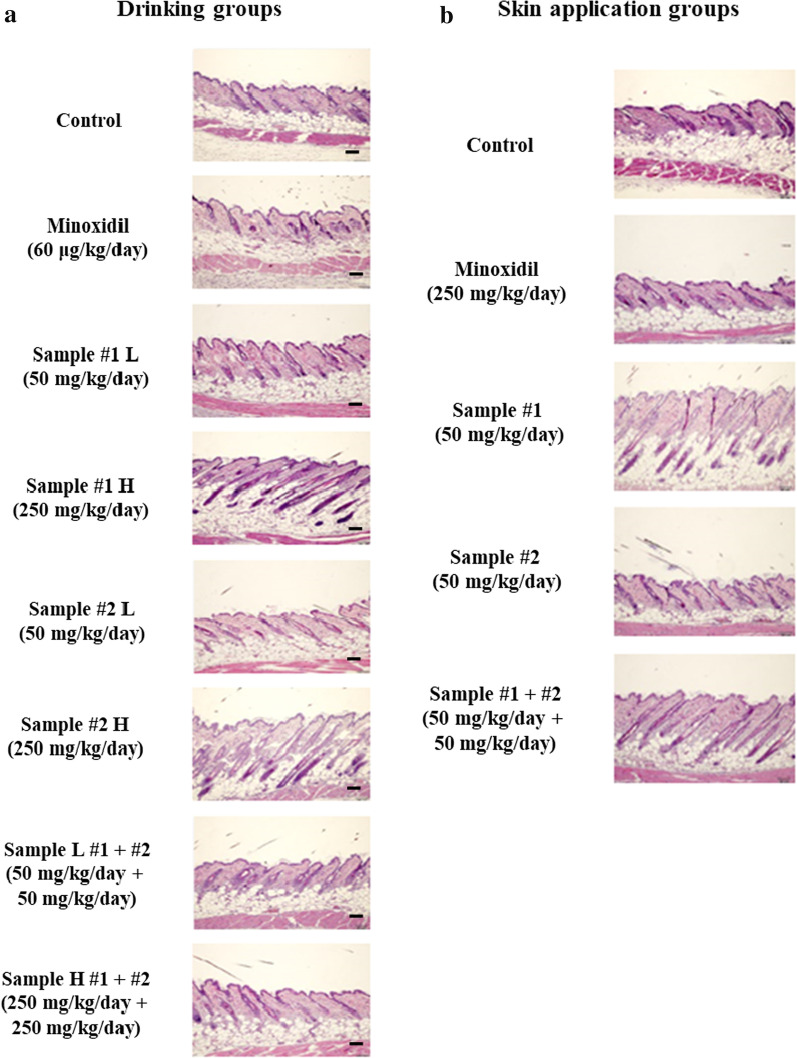
Histological examinations of the dorsal skin of *C. tricuspidata* and/or *S. fusiforme* treated C57BL/6 mice. Hematoxylin and eosin staining of the dorsal skin of mice in the drinking groups **a** and skin application groups (**b**). Mice were treated with either *C. tricuspidata* or *S. fusiforme* or their combination*,* minoxidil (positive drug group), and drinking water. Images were acquired using a microscope at 100 $\times$ magnification; scale bars = 100 μm. The most representative observations are shown in this Figure. Sample #1: *C. tricuspidata* and Sample #2: *S. fusiforme*. Doses of samples were applied to groups, as described in the Materials and methods section; L: Low dose and H: High dose

**Fig. 4:**
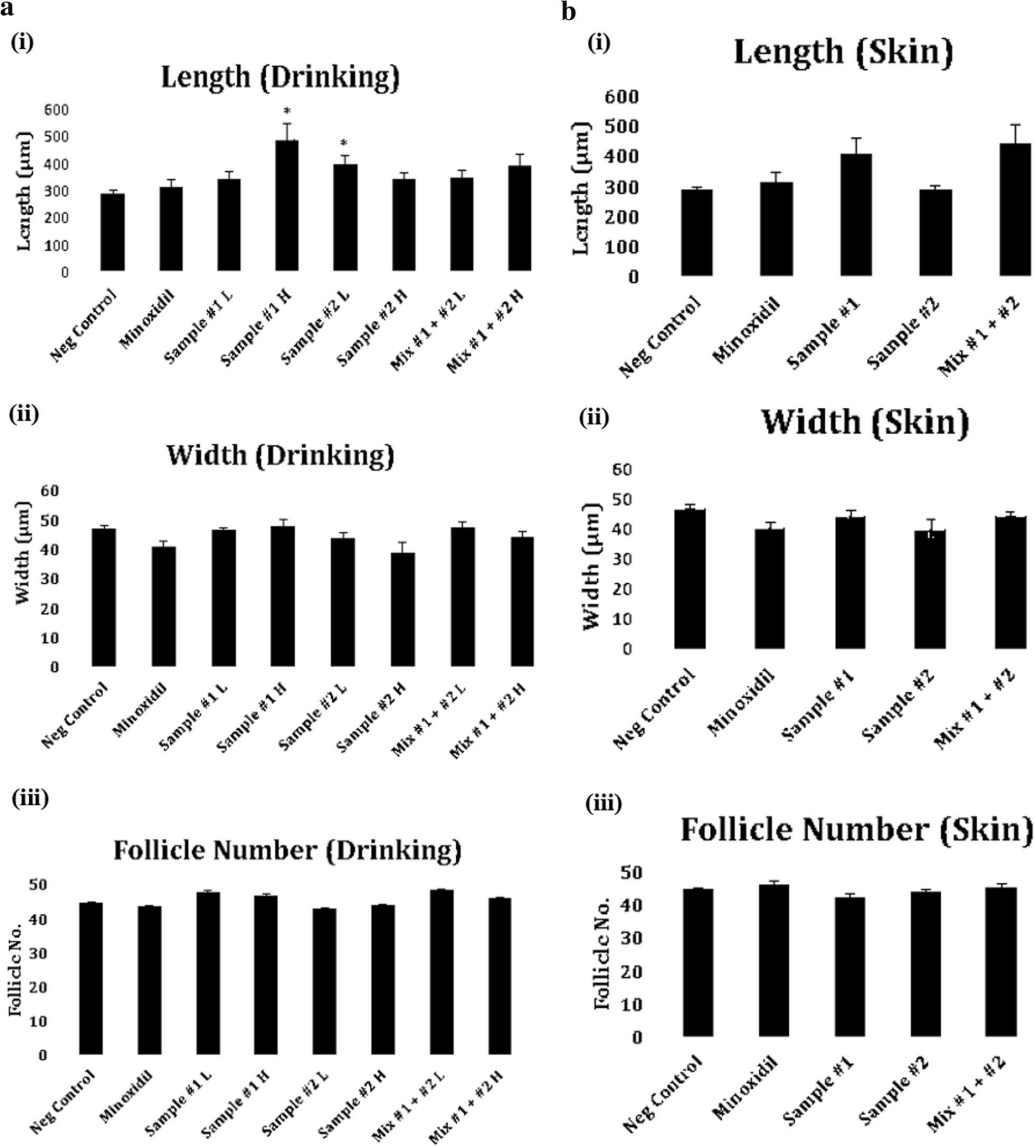
Effects of *C. tricuspidata* and/or *S. fusiforme* on hair follicles number and size in mice. Based on histological examination of the dorsal skin of mice, the length, width, and the number of hair follicles in the drinking group **a** and the skin application group **b** treated with *C. tricuspidata* and/or *S. fusiforme* were measured on day 22. Data are presented as mean ± SEM. ^*^p < 0.05 compared with the control group. Sample #1: *C. tricuspidata* and Sample #2: *S. fusiforme*. Doses of samples were applied to groups, as described in the Materials and methods section; L: Low dose and H: High dose

The effects of *C. tricuspidata* and *S. fusiforme* on the size and density of hair follicles in the dorsal skin of C57BL/6 mice were evaluated using hematoxylin and eosin staining on day 22 (Fig. 3). In the drinking groups, the *C. tricuspidata* (higher dose) and *S. fusiforme* (lower dose) groups showed significantly longer (*p* < 0.05) hair follicles than the control group (Fig. 4a (i)). In the drinking groups, even though the apparent hair growth intensities are not different between the C. tricuspidata (higher dose) and C. tricuspidata (lower dose) groups (Figs. 1 and 2), the length of hair follicles in the group treated with high dose of C. tricuspidata showed significantly higher value compared with the value in the group treated with low dose of C. tricuspidata at day 22 (Fig. 4). In the skin application groups, the mice in *C. tricuspidata* and *C. tricuspidata* + *S. fusiforme* subgroups showed longer hair follicles than that in control subgroup mice; however, no significant difference was observed (Fig. 4b (i)). None of the treatments showed significant effects on the width and number of follicles compared to the no-treatment control (Fig. 4a (ii–iii), b (ii–iii)).

### *C. tricuspidata* and *S. fusiforme* extracts upregulated genes related to anagen factors

The differential expression of genes related to anagen factors in the dorsal skin of C57BL/6 mice was evaluated using RNA sequencing. In the drinking groups, the expression of genes related to several anagen factors was increased compared to their expression in the control group (Table 1). In particular, *C. tricuspidata* administered via drinking upregulated (> twofold) *Ctnnbl1*, *Pdgf, Vegf*, and *Wnt* in treated mice compared to that in mice without the supplementation of *C. tricuspidata* extracts (Table 2). In contrast, only *Vegf* and *Wnt* were upregulated (> twofold) in the *S. fusiforme* drinking subgroup compared to that in the control subgroup.

**Table 1:**
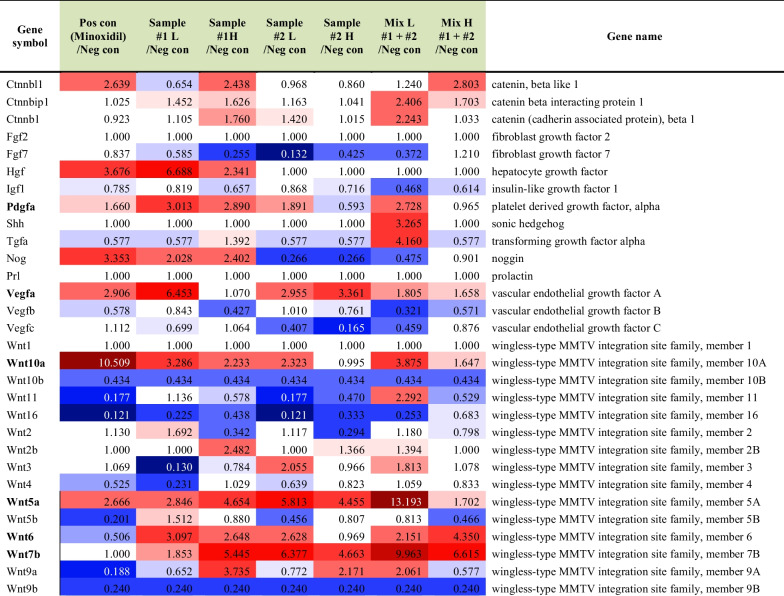
Differential gene expression analysis of genes related to anagen factors in the *C. tricuspidata* and/or *H. fusiforme* drinking groups

#1; *C. tricuspidata* and #2; *S. fusiforme*

**Table 2:** Up-regulated (> twofold) genes related to anagen factors in the *C. tricuspidata* and/or *H. fusiforme* drinking groups

Gene symbol	Minoxidil /Control	#1 L /Control	#1 H /Control	#2 L /Control	#2 H /Control	Mix L #1 + #2 /Control	Mix H #1 + #2 /Control
Ctnnbl1	2.639		2.438				2.803
Pdgfa		3.013	2.890			2.728	
Vegfa	2.906	6.453		2.955	3.361		
Wnt10a	10.509	3.286	2.233	2.323		3.875	
Wnt5a	2.666	2.846	4.654	5.813	4.455	13.193	
Wnt6		3.097	2.648	2.628		2.151	4.350
Wnt7b			5.445	6.377	4.663	9.963	6.615

#1; *C. tricuspidata* and #2; *S. fusiforme*

In the skin application groups, the expression levels of genes related to several anagen factors were increased compared to the control group by application of *C. tricuspidata* and/or *H. fusiforme* (Table 3). Particularly, *C. tricuspidata* skin application groups showed up-regulation (> twofold) of genes related to anagen factors including β-Catenin (*Ctnnbl1*), *Pdgf*, *Tgf*, *Vegf*, *Wnt5a*, and *Wnt7b* genes compared to the control group (Table 4). On the other hand, *H. fusiforme* skin application groups up-regulated only *Tgf*, *Vegf*, and *Wnt7b* genes compared to the control group. The results confirmed that both *β-Catenin* and *Pdgf* genes are up-regulated only by *C. tricuspidata* whereas *Vegf* and *Wnt*s genes are up-regulated by either *C. tricuspidata* or *H. fusiforme* in the dorsal skin of C57BL/6 mice.

**Table 3:**
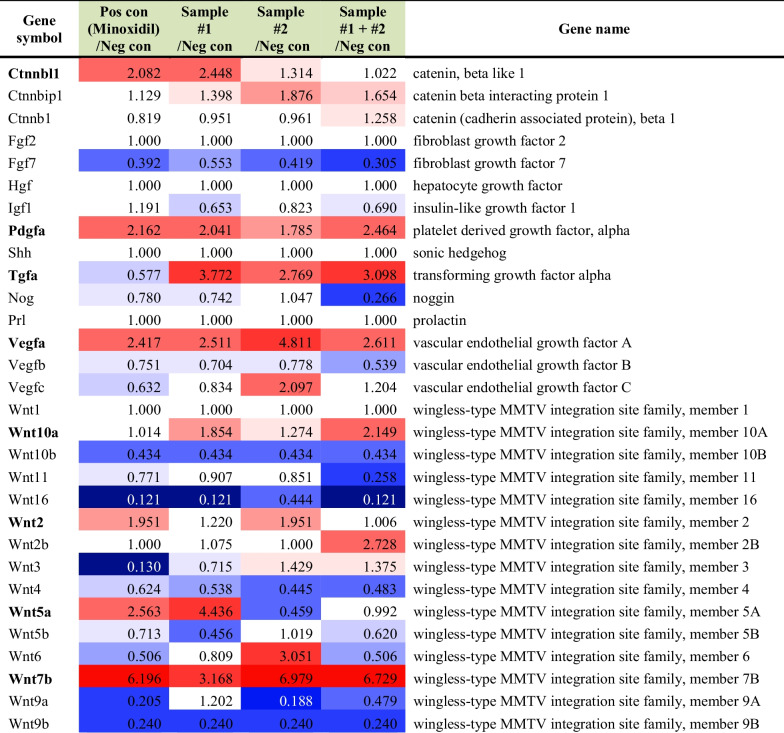
Differential gene expression analysis of genes related to anagen factors in the *C. tricuspidata* and/or *H. fusiforme* skin application groups

#1; *C. tricuspidata* and #2; *S. fusiforme*

**Table 4:** Up-regulated (> twofold) genes related to anagen factors in the *C. tricuspidata* and/or *H. fusiforme* skin application groups

Gene symbol	Minoxidil /Control	#1 /Control	#2 /Control	#1 + #2 /Control
Ctnnbl1	2.082	2.448		
Pdgfa	2.162	2.041		2.464
Tgfa		3.772	2.769	3.098
Vegfa	2.417	2.511	4.811	2.611
Wnt5a	2.563	4.436		
Wnt7b	6.196	3.168	6.979	6.729

#1; *C. tricuspidata* and #2; *S. fusiforme*

### *C. tricuspidata* and *S. fusiforme* extracts upregulated genes related to catagen-telogen factors

The RNA sequencing analysis revealed that drinking *C. tricuspidata* or *S. fusiforme* extracts downregulated several catagen-telogen factor-related genes compared to those without supplements (the control subgroup) (Table 5). In *C. tricuspidata* drinking groups, genes related to catagen-telogen factors, including *Il1β* and *Osm,* were downregulated (< 0.5 fold) compared to those in the control group. S*. fusiforme* drinking groups showed downregulation of *Bdnf* and *Osm* compared to the control group (Table 6). In the skin application group, the expression of *Bdnf* and *Osm* was decreased in *C. tricuspidata* and/or *S. fusiforme* subgroups compared to that in the control group (Tables 7, 8).

**Table 5:**
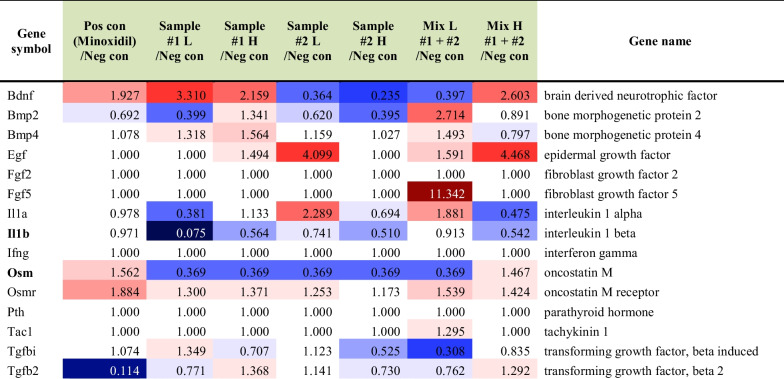
Differential gene expression analysis of genes related to catagen-telogen factors in the *C. tricuspidata* and/or *H. fusiforme* drinking groups

#1; *C. tricuspidata* and #2; *S. fusiforme*

**Table 6:** Down-regulated (< 0.5 fold) genes related to catagen-telogen factors in the *C. tricuspidata* and/or *H. fusiforme* drinking groups

Gene symbol	Minoxidil /Control	#1 L /Control	#1 H /Control	#2 L /Control	#2 H /Control	Mix L #1 + #2 /Control	Mix H #1 + #2 /Control
Bdnf				0.364	0.235	0.397	
Il1b		0.075					
Osm		0.369	0.369	0.369	0.369	0.369	

#1; *C. tricuspidata* and #2; *S. fusiforme*

**Table 7:**
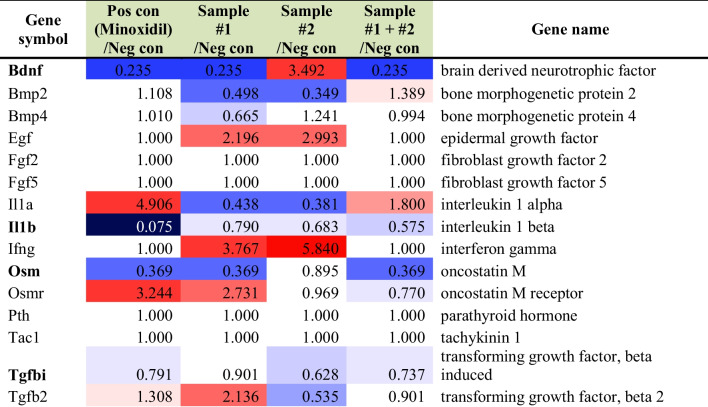
Differential gene expression analysis of genes related to catagen-telogen factor in the *C. tricuspidata* and/or *H. fusiforme* skin application groups

#1; *C. tricuspidata* and #2; *S. fusiforme*

**Table 8:** Down-regulated (< 0.5 fold) genes related to catagen-telogen factors in the *C. tricuspidata* and/or *H. fusiforme* skin application groups

Gene symbol	Minoxidil /Control	#1 /Control	#2 /Control	#1 + #2 /Control
Bdnf	0.235	0.235		0.235
Il1b	0.075			
Osm	0.369	0.369		0.369

#1; *C. tricuspidata* and #2; *S. fusiforme*

## Discussion

The present study demonstrated that drinking and skin application of *C. tricuspidata* and/or *S. fusiforme* increased the hair growth rate in the dorsal skin of C57BL/6 mice. In addition, *C. tricuspidata* increased follicle length in the dorsal skin of C57BL/6 mice in both drinking and skin application groups. RNA sequencing analysis revealed that β-catenin and *Pdgf* genes were upregulated only by *C. tricuspidata*, whereas *Vegf* and *Wnts* genes were upregulated by either *C. tricuspidata* or *S. fusiforme* in the dorsal skin of C57BL/6 mice. Furthermore, RNA sequencing revealed that both drinking and skin application of *C. tricuspidata* downregulated the expression of *Osm* in the dorsal skin of C57BL/6 mice.

A previous study has shown that β-catenin is involved in forming placodes that generate hair follicles in mice [12, 13]. *Pdgf* contributes to the formation of dermal papillae, which plays an important role in the regulation of hair growth [14, 15]. Overexpression of *Vegf* induces perifollicular vascularization, resulting in accelerated hair regrowth and increased size of hair follicles in mice [16]. Wnt signaling plays a key role in stimulating hair follicle stem cells and hair regeneration in mice [17, 18]. Taken together, the present study confirms that drinking and skin application of *C. tricuspidata* and/or *S. fusiforme* extracts enhanced hair growth by upregulating the expression of anagen factors in C57BL/6 mice.

The results also showed that both drinking and skin application of *C. tricuspidata* downregulated *Osm* in C57BL/6 mice. *Osm* is a negative regulator of hair growth, and its overexpression leads to hair loss in mice [19, 20]. Therefore, we inferred that *C. tricuspidata* and/or *S. fusiforme* extracts also downregulate the negative regulators of hair growth. Zhu et al. [21] established the hair loss C57BL/6 mouse model by inducing with DHT (dihydrotestosterone). In that study, the application of Serenoa repens extract promoted hair regeneration in hair loss mouse model by activity TGF-β signaling and mitochondrial signaling pathway. In the present study, authors performed RNA sequencing analysis to screen the differential expression of genes related to anagen factors from 32,000 genes in mouse library. Even though the present study observed only the screened gene expression here, our future study will examine the differential expression of proteins to explain the mechanism of anagenic effects of C. tricuspidata and S. fusiforme extracts.

## Conclusions

The present study reported that drinking and application of C*. tricuspidata* or *S. fusiforme* upregulated the genes regulating the anagen phase of hair growth, including β-catenin, *Pdgf, Vegf,* and *Wnts*, and downregulated the genes associated with catagen-telogen phases, such as *Osm*, consequently leading to increased hair follicle length and a higher hair growth rate in the dorsal skin of C57BL/6 mice. Collectively, these results suggest that *C. tricuspidata* and *S. fusiforme* extracts are potential candidates for the development of therapeutics to treat hair loss. However, large-scale human clinical trials to establish the safety and efficacy of *C. tricuspidata and S. fusiforme* may lead to the development of effective therapy for hair loss treatment in the future.

## Methods

### Materials

Minoxidil (5 mg tablet) was purchased from Hyundai Pharmaceutical Company (Korea), and minoxidil (5% liquid) was from Dongkook Pharmaceutical Co., Ltd. (Korea). Hair removal cream (NairTM) was purchased from Church & Dwight Co., Inc. (USA).

### Animals

Six-week-old female C57BL/6 mice were obtained from Orient Bio (Korea) and allowed to adapt for a week with food and water ad libitum. Mice were housed under controlled temperature (24 °C), humidity (50%–55%), and photoperiod (12 h light:12 h darkness cycle) in the animal facility at Jeju National University. All experiments were carried out in accordance with the National Institute of Health Guide for the Care and Use of Laboratory Animals and were approved by the Institutional Animal Care and Use Committee of Jeju National University (approval number: 2022–0003).

### Preparation of the extracts

The extracts of C. tricuspidata (BK-CTE 50) and H. fusiforme (BK-HFE 50) were prepared at BK bio (Korea). Briefly, raw materials of C. tricuspidata and S. fusiforme were extracted with 50% ethanol and concentrated by vacuum evaporator. The concentrates were sterilized, dried and stored at cool temperature before use.

### Experimental design

C57BL/6 mice were divided into two groups: drinking (*n* = 72) and skin application (*n* = 45) based on the mode of treatment. In the drinking group, the mice were randomly divided into eight subgroups (*n* = 9): control, minoxidil (60 μg/kg/day), *C. tricuspidata*-lower dose (50 mg/kg/day), *C. tricuspidata* higher dose (250 mg/kg/day), *S. fusiforme*-lower dose (50 mg/kg/day), *S. fusiforme*-higher dose (250 mg/kg/day), a mixture of *C. tricuspidata* and *S. fusiforme*-lower dose (50 mg/kg/day + 50 mg/kg/day), and a mixture of *C. tricuspidata* and *S. fusiforme*-higher dose (250 mg/kg/day + 250 mg/kg/day). In the skin application group, mice were divided into five subgroups (*n* = 9): control, minoxidil (250 mg/kg/day), *C. tricuspidata* (50 mg/kg/day), *S. fusiforme* (50 mg/kg/day)*,* and a mixture of *C. tricuspidata* and *S. fusiforme* (50 mg/kg/day + 50 mg/kg/day)*.* Before the application of the designated medication, hair from the dorsal skin of mice in both groups was shaved using a clipper, and hair removal cream was applied externally at 7 weeks of age, by which all follicles were synchronous in the anagen stage. For the drinking groups, appropriate amounts of respective medications were dissolved in drinking water and administered to each group for 21 days, assuming that each mouse (average body weight is 20 g) drinks 5 mL water daily. For the skin application group, 100 µL of the indicated medications were applied topically to the dorsal skin of each mouse once daily for 21 days.

### ImageJ analysis

ImageJ analysis was used to analyze the hair growth rate in the drinking and the skin application groups. During the experiment, images were captured from days 5 to 21 at an interval of 2 days. Image J analysis was performed to estimate the hair growth rate in the dorsal skin of the mice in each group. The hair growth rate was estimated using the following equation: [(Intensity on day 1—Intensity on a respective day)/Intensity on day 1] $\times$ 100.

### Histological analysis

Histological analysis was performed to measure the length, width, number, and density of hair follicles in the drinking and skin-application groups. At the end of the experiment (day 22), the dorsal skin of each mouse in the drinking and skin application groups was surgically removed for histological analysis. The excised skin specimens were immediately fixed in 10% formalin, processed routinely, and embedded in paraffin blocks to prepare the tissue sections. Skin sections were stained with hematoxylin and eosin, and the length, width, number, and density of hair follicles were measured in the drinking and skin application groups.

### RNA sequencing analysis

At the end of the experiment (day 22), the dorsal skin of each mouse in the drinking and skin application groups was surgically removed for RNA sequencing analysis, which was performed as described in previous studies [22–24]. Total RNA was isolated from dorsal skin tissues using an Easy-Blue RNA Extraction Kit (iNtRON Biotechnology, Korea). RNA quality was assessed using an Agilent 2100 Bioanalyzer and RNA 6000 Nano Chip (Agilent Technologies, Netherlands). Based on the manufacturer’s instructions, RNA libraries were constructed using the Quantseq 3ʹ mRNA-Seq Library Prep Kit (Lexogen, Austria). High-throughput sequencing was performed as single-end 75 sequencing using a NextSeq 500 (Illumina, San Diego, CA, USA). QuantSeq 3ʹ mRNA-Seq reads were aligned using Bowtie2 version 2.1.0 [25]. Differentially expressed genes were determined based on counts from unique and multiple alignments using EdgeR in R version 3.2.2 and Bioconductor version 3.0 [26]. The read count data were processed based on the quantile normalization method using GenowizTM version 4.0.5.6 (Ocimum Biosolutions, Hyderabad, India). Gene classification was performed using the Medline database (National Center for Biotechnology Information, USA).

### Statistical analysis

Data are expressed as mean ± standard error of the mean (SEM) of nine replications. All statistical analyses were performed using IBM SPSS Statistics (Ver.17.0; USA). Statistical differences among groups were analyzed using one-way analysis of variance (ANOVA), followed by Tukey’s test. Differences were considered statistically significant at *p* < 0.05.

## Abbreviations

Bdnf: Brain derived neurotrophic factor
Ctnnb1: Catenin Beta 1
H: High dose
Il1β: Interleukin 1 Beta
L: Low dose
Osm: Oncostatin M
Pdgf: Platelet-derived growth factor
TGF: Transforming growth factor
TGF-β/BMP: Transforming growth factor-β/bone morphogenic protein
Vegf: Vascular endothelial growth factor
Wnts: Wingless-related integration site
Wnt5a: Wnt family member 5A
Wnt7b: Wnt family member 7B
